# Topoisomerase-Mediated DNA Damage in Neurological Disorders

**DOI:** 10.3389/fnagi.2021.751742

**Published:** 2021-11-25

**Authors:** Morgan Crewe, Ram Madabhushi

**Affiliations:** Departments of Psychiatry, Neuroscience, and Cell Biology, University of Texas Southwestern Medical Center, Dallas, TX, United States

**Keywords:** topoisomerase, DNA cleavage complexes, TDP1, TDP2, neurodegeneration

## Abstract

The nervous system is vulnerable to genomic instability and mutations in DNA damage response factors lead to numerous developmental and progressive neurological disorders. Despite this, the sources and mechanisms of DNA damage that are most relevant to the development of neuronal dysfunction are poorly understood. The identification of primarily neurological abnormalities in patients with mutations in TDP1 and TDP2 suggest that topoisomerase-mediated DNA damage could be an important underlying source of neuronal dysfunction. Here we review the potential sources of topoisomerase-induced DNA damage in neurons, describe the cellular mechanisms that have evolved to repair such damage, and discuss the importance of these repair mechanisms for preventing neurological disorders.

## Introduction

Eukaryotic genomes are highly organized and hierarchically folded to fit within a relatively tiny nucleus. Achieving the necessary compaction (∼ 10^4^–10^5^ fold for mammalian cells) while preserving access to genetic information requires DNA supercoiling, which causes DNA to become either overwound or underwound relative to its normal helical pitch (Vologodskii et al., 1992; Holmes and Cozzarelli, 2000; Baranello et al., 2012; Corless and Gilbert, 2016). Supercoiling injects free-energy into DNA molecules that can influence various cellular processes—underwound DNA favors helix opening during transcription and DNA replication and stabilizes DNA-protein interactions, whereas DNA-protein contacts are destabilized in overwound DNA (Holmes and Cozzarelli, 2000; Corless and Gilbert, 2016). Classical studies have shown that the torque generated from DNA unwinding during the movement of RNA and DNA polymerases is the primary source of DNA supercoiling within eukaryotic cells (Liu and Wang, 1987; Baranello et al., 2012; Corless and Gilbert, 2016). Torsional stress from DNA supercoiling is, in turn, relieved through the actions of DNA topoisomerases.

In mammalian cells, the DNA topoisomerases, TOP1, TOP2A, and TOP2B chiefly resolve torsional stress from DNA supercoiling. DNA topoisomerases create transient DNA breaks and pass DNA strands through each other, and in so doing, solve various topological problems that arise during transcription, DNA replication, and chromosome segregation (Wang, 2002; Vos et al., 2011; Pommier et al., 2016). Although the formation of a DNA break is a necessary and typically transient event, it is now well understood that several mechanisms exist to prevent religation of the DNA break, resulting in long-lasting DNA damage that requires activation of the DNA damage response for repair. Therefore, even though these enzymes are essential for cell survival, topoisomerases also have the capacity to damage the genome through their normal catalytic activity. The consequences of topoisomerase-mediated DNA damage have been extensively studied in the context of DNA replication and chromosome segregation and have been exploited to develop important anticancer and antimicrobial drugs (Pommier et al., 2010; Pommier and Osheroff, 2012). However, until recently, there has been a surprising lack of focus on the most prominent victims of topoisomerase-mediated DNA damage-post—mitotic neurons. Mutation to enzymes responsible for the repair of topoisomerase-mediated DNA damage results in nearly exclusively neurological phenotypes, but it remains unclear how disruption of a process that is crucial for the survival of all cells can be selectively toxic to post-mitotic neurons. Neurons present a unique topological context that is seldom encountered in other cell types and express a distinct set of topoisomerases compared to dividing cells (McKinnon, 2016; Madabhushi, 2018). Post-mitotic neurons are free of the topological challenges that arise from DNA replication and cell cycle transitions, but are also some of the longest living, most dynamic cells that must contend with the cumulative effects of basal and stimulus-dependent transcription.

The recent identification of neurological disorders with causal mutations in enzymes that specialize in the repair of topoisomerase-mediated DNA damage calls for an examination of the contribution of these lesions to neurological disease (Zagnoli-Vieira and Caldecott, 2020). In this review, we discuss the causes of topoisomerase-mediated DNA damage that could be pertinent to genome integrity in neurons, how these lesions are repaired, and the significance of defective repair of topoisomerase-generated DNA damage to the development of neurological disorders.

## Sources of Topoisomerase-Induced DNA Damage in Neurons

Topoisomerases catalyze the formation of transient single-strand (TOP1) or double-strand (TOP2A and TOP2B) DNA breaks that are usually re-ligated by the enzyme itself. A key step in this catalytic cycle is the formation of a transient cleavage complex intermediate (TOPcc) involving a covalent bond between the topoisomerase active site tyrosine and the DNA end (Wang, 2002). Under certain conditions, however, the intermediate cleavage complex is stabilized on the DNA, preventing religation by the enzyme and triggering a DNA damage response. Historically, topoisomerase-mediated DNA breaks were thought of as failed reaction intermediates that occur randomly and infrequently and were not thought to be a major source of DNA damage in mammalian cells. However, two recent sources of evidence have challenged these ideas. First, evidence of accumulating topoisomerase-mediated DNA damage has been observed in a number of neurological disorders caused by mutation to DNA repair factors, suggesting endogenous topoisomerase activity is a major source of DNA damage in mammalian cells. Second, recent studies show that topoisomerase-mediated DNA breaks can be formed in direct response to physiological stimuli to facilitate cell-type specific gene expression programs, suggesting topoisomerase-mediated DNA breaks are not only a frequent event but can serve important functional roles in the cell. Understanding how these long-lasting DNA breaks form could prove extremely valuable to our understanding of neurobiology and DNA damage related disease pathology.

**FIGURE 1:**
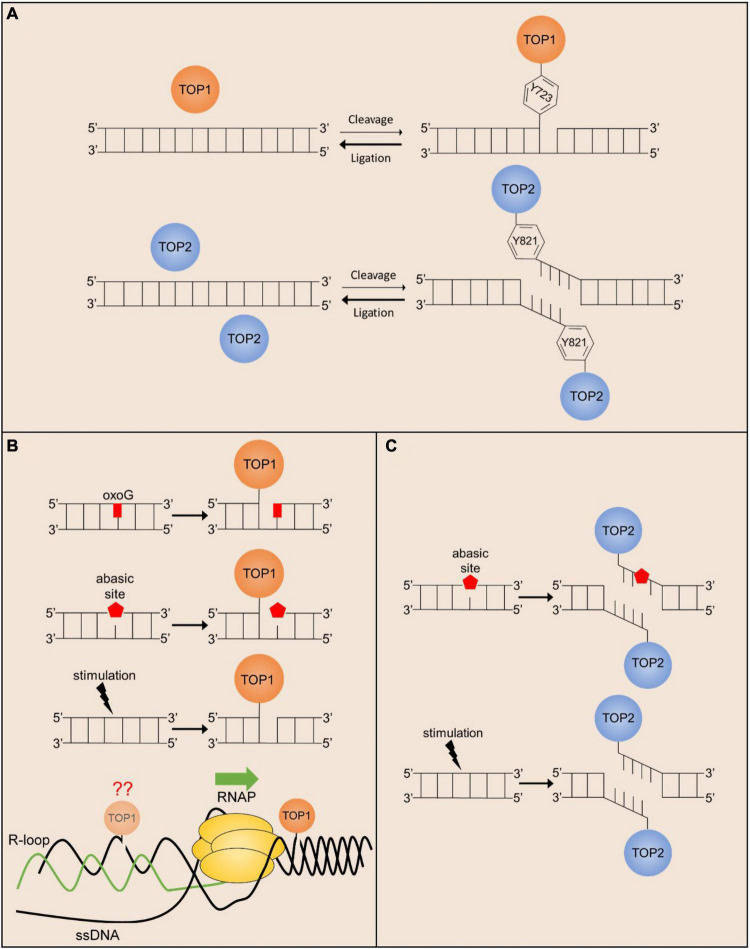
Endogenous mechanisms of stalled TOP1 and TOP2 cleavage complex formation. **(A)** The cleavage/ligation reaction of TOP1 (monomer) and TOP2 (homodimer) greatly favors ligation over cleavage. Active site amino acid positions are given for hTOP1 and hTOP2B. **(B)** TOP1cc religation is prevented in the presence of oxidated bases and abasic sites, and the formation of TOP1-mediated SSBs can be facilitated in response to ligand-dependent stimulation. TOP1 acting in front of the polymerase can also stall transcription promoting R-loop formation, but TOP1 may also act behind the polymerase where R-loop formation can prevent TOP1cc religation. **(C)** TOP2cc religation is prevented in the presence of abasic sites, and the formation of TOP2-mediated DSBs can be facilitated in response to cell stimulation.

A number of sources of topoisomerase-induced DNA lesions have been described and reviewed previously (Pommier et al., 2016). Notably, however, the significance of these mechanisms for topoisomerase-induced DNA damage in neurons remains largely uninvestigated and therefore only a few potentially relevant examples are considered here (Figure 1). As mentioned above, the DNA cleavage-religation reaction cycle of topoisomerases has been exploited to develop potent chemotherapeutic drugs. A majority of these drugs work by trapping either topoisomerase I (TOP1; Camptothecin, Topotecan, and Irinotecan) or topoisomerase II (TOP2A and TOP2B; Etoposide, Teniposide, and Doxorubicin) in enzyme-mediated DNA cleavage complexes that get converted into cytotoxic DNA single strand breaks (SSBs) and double strand breaks (DSBs) and induce the apoptosis of cancer cells. Although chemotherapy is lifesaving for many cancer patients, chemotherapeutic drugs also cause widespread topoisomerase-induced DNA damage in non-cancer cells and tissues. Chemotherapy-related cognitive impairments (CRCI), including diminished verbal ability, working memory, and executive function have been extensively documented in cancer survivors that receive Doxorubicin treatments (El-Agamy et al., 2019). Rodent models treated with Doxorubicin or Topotecan also display defects in spatial and associative learning (Seigers et al., 2015; El-Agamy et al., 2019; Matsos and Johnston, 2019; Nguyen and Ehrlich, 2020). Together, these studies indicate that topoisomerase poisons used in chemotherapy could be relevant sources of DNA damage in the nervous system. Despite this, the precise mechanisms by which chemotherapy utilizing topoisomerase poisons cause cognitive deficits are unknown. For instance, whereas TOP2 trapping and cytotoxic DSB formation explains how Doxorubicin kills various cancer cells, early studies indicated that systemically administered Doxorubicin does not cross the blood-barrier in doses known to kill tumor cells (Bigotte et al., 1982; Tangpong et al., 2006). A thorough examination of whether the low doses of Doxorubicin and other topoisomerase poisons that do cross the blood brain barrier are sufficient to poison TOP2 in the brain should help address this issue.

The discovery that the DNA cleavage-religation cycle of topoisomerases could be poisoned by exogenous agents led to investigations into whether topoisomerase-mediated DNA cleavage could also be enhanced by endogenous cellular factors. Early *in vitro* studies identified that a variety of DNA lesions, such as abasic sites, oxidative base damage, and bulky adducts markedly stimulate DNA cleavage by TOP1, TOP2A, and TOP2B. For instance, studies examining the activity of purified recombinant TOP2A and TOP2B on plasmid DNA containing randomly incorporated abasic sites indicated that TOP2 isoforms can locate sparse lesions within thousands of undamaged base pairs, and that even a few lesions can markedly stimulate TOP2-mediated DNA cleavage (Sabourin and Osheroff, 2000). Similarly, bulky lesions also stimulated TOP2-mediated DNA cleavage whereas oxidative lesions, such as 8-oxoguanine, which do not distort the double helix, had little impact on DNA scission by TOP2A and TOP2B (Sabourin and Osheroff, 2000). By contrast, 8-oxoguanine was shown to be a potent TOP1 poison (Pourquier et al., 1999; Lesher et al., 2002).

The potential importance of these *in vitro* finding can immediately be understood in the context of post-mitotic neurons. Abasic sites and oxidized bases are some of the most commonly formed DNA lesions in cells, ranging between 50,000 and 200,000 per genome, but basal levels of these lesions are found to be the greatest in the brain compared to other organs (Nakamura and Swenberg, 1999). The high load of mitochondria found in neurons produces high levels of reactive oxygen species that generate thousands of oxidative lesions per day (Narciso et al., 2016). Treatment of cells with exogenous agents that induce oxidative DNA damage have been shown to stabilize TOP1 and TOP2ccs (Li et al., 1999; Daroui et al., 2004; Sordet et al., 2004), suggesting that oxidative lesions could be a major source of topoisomerase poisoning in the nervous system. However, the extent to which such trapping occurs from the actions of physiologically generated reactive oxygen species is not properly understood. Recent genome-wide analysis of TOP1 and TOP2 occupancy patterns suggest that topoisomerase activity is tightly regulated within the genome (Madabhushi et al., 2015; Baranello et al., 2016; Gittens et al., 2019). A direct comparison to determine whether topoisomerase occupancy is enriched at genome-wide hotspots for accumulation of abasic sites and oxidative DNA damage in neurons should provide insights into these matters.

Whereas endogenous and exogenous agents can stabilize TOP1 and TOP2 ccs, some studies have shown that this event can be exacerbated by additional endogenous processes. For instance, Camptothecin (CPT) treatment induces DSBs, elevated p53 levels, and cytotoxicity in a replication-dependent manner (Hsiang et al., 1989; D’Arpa et al., 1990; Ryan et al., 1991; Pommier, 2006). These studies suggest that TOP1ccs can be converted into other cytotoxic lesions through interactions with other cellular processes, such as DNA replication and transcription (Pommier, 2006; Pommier et al., 2016). Because neurons are post-mitotic, it was initially speculated that they could be more resistant to CPT. However, CPT treatment in neurons also induces DSBs and apoptosis in a dose-dependent manner that can be prevented in the presence of transcription inhibitors (Morris and Geller, 1996; Sordet et al., 2009). These results suggest that ongoing transcription could also act upon trapped TOP1ccs and induce the formation of DNA DSBs. However, the precise roles conditions under which transcription machinery is most likely to encounter and process TOP1ccs are not fully understood, and many possibilities have been proposed. On the one hand, it is possible that the collision of frozen TOP1 with transcribing RNA polymerases induces the formation of DNA DSBs. Interestingly, however, DSB formation by CPT could be suppressed by the overexpression of RNase H, which primarily resolves R-loops that form between the nascent RNA transcript and the template DNA strand (Sordet et al., 2009). These results suggest an alternative possibility in which increased levels of underwound DNA in the presence of TOP1 inhibitors cause the formation R-loops, that in turn, lead to the formation of DSBs through yet unidentified mechanisms. Interestingly, analysis of repair-deficient models (see below) indicate that TOP1-induced DNA damage accumulates within the brain under physiological conditions. Because TOP1 is primarily active within transcriptionally active regions (Baranello et al., 2016), these results suggest that interaction with the transcription machinery could be an important potential source of TOP1-induced DNA damage under physiological conditions in neurons.

The sources described so far involve the accidental poisoning of TOP1 and TOP2 cleavage complexes in response to endogenous and exogenous agents, but more recent studies suggest that TOP1 and TOP2-mediated DNA breaks can directly and reproducibly be induced following cellular stimulation. Several studies have now shown that stimulus-responsive genes in a variety of cell types and systems, including those that are induced upon exposure to estrogen, insulin, glucocorticoids, and serum, incur TOP2B-mediated DSBs within their promoters (Ju et al., 2006; Wong et al., 2009; Haffner et al., 2010; Bunch et al., 2015; Trotter et al., 2015). Similarly, *in vitro* stimulation of primary cortical neurons or *in vivo* exposure to associative learning tasks induce the formation of TOP2B-mediated DSBs (Suberbielle et al., 2013; Madabhushi et al., 2015). These DSBs are distinct from the transient DNA breaks created by TOP2B during its normal catalytic cycle in that the formation of TOP2B-mediated DSBs activates the cellular DNA damage response and the formation of γH2AX (Bunch et al., 2015; Madabhushi et al., 2015; Stott et al., 2021). Furthermore, stimulus-dependent DSBs generated by TOP2B are repaired using classical DSB repair pathways, such as non-homologous end joining (NHEJ) in neurons (Madabhushi et al., 2015). Activity-induced DSBs are not randomly distributed throughout the genome, but rather occur within the promoters of a subset of neuronal activity-responsive genes known as early response genes (ERGs) (Madabhushi et al., 2015). Surprisingly, the formation of TOP2B-mediated DSBs facilitates the rapid stimulus-dependent transcription of ERGs (Madabhushi et al., 2015). Neuronal ERGs are enriched for transcription factors, such as *Fos*, *Npas4*, *Egr1*, *FosB*, *Nr4a1*, and *Nr4a3*, that facilitate the expression of various late-response genes. Ultimately, ERGs mediate experience-driven changes to synapses and neural circuits that underlie the development of lasting behavioral adaptations such as learning and long-term memory formation (West and Greenberg, 2011; Madabhushi and Kim, 2018; Yap and Greenberg, 2018).

These results are intriguing because they imply that the formation of lasting DSBs by TOP2B serves a physiological function in the transcription of stimulus-responsive genes. For instance, whereas knockdown of *Top2b* attenuated the expression of neuronal ERGs, generating DSBs within ERG promoters using CRISPR was able to restore ERG expression under these conditions, suggesting that DSB formation plays a key role in the transcriptional induction of ERGs (Madabhushi et al., 2015). It is still unclear whether the formation of lasting TOP2B-mediated DSBs in this manner constitutes a novel mechanism of topoisomerase regulation or whether it represents yet another mechanism of accidental topoisomerase trapping. Addressing this issue requires a deeper understanding of how lasting TOP2B-mediated DSBs are orchestrated. Analysis of TOP2B occupancy patterns in neurons using ChIP-seq has provided some insights into this issue (Madabhushi et al., 2015). First, TOP2B binding is only enriched at a few hundred sites within the genome of neurons, suggesting that its activity could be tightly regulated. Second, whereas neuronal stimulation caused a 4–5-fold increase in TOP2B occupancy within the genome, neuronal activity-induced DSBs were not detected at these sites. Instead, DSB formation was specifically detected only at a small subset of sites containing pre-bound TOP2B in unstimulated neurons. These results suggest that TOP2B binding alone is not sufficient for DSB formation, and that its activity is somehow modulated to induce lasting DSBs at certain genomic sites. Furthermore, whereas ERGs are rapidly and highly induced following neuronal stimulation, inhibiting transcriptional elongation does not prevent the formation of TOP2B-mediated DSBs following neuronal activity (Madabhushi et al., 2015). By contrast, the escape of promoter-proximally paused RNA polymerase II (RNAPII) was reduced following TOP2 inhibition (Bunch et al., 2015). Together, these results suggest that TOP2B-mediated DSBs are unlikely to generated as a byproduct of transcription elongation at these sites. Interestingly, recent findings suggest that posttranslational modification of TOP2 could affect its DNA cleavage activity (Bedez et al., 2018; Vanden Broeck et al., 2021). While these studies were conducted for TOP2A, similar investigations into how signaling events triggered in response to neuronal stimulation modify TOP2B could provide much needed insights in this area. In addition to TOP2B-mediated DSBs, TOP1-mediated SSBs are shown to promote ligand-dependent enhancer activation, though the full significance of this observation has not yet been elucidated (Puc et al., 2015).

The link between TOP2B-mediated DSBs and stimulus-dependent gene transcription further suggests that changes in either the formation or repair of activity-induced DSBs could impact neuronal functions. These results also underscore the importance of efficient repair of topoisomerase-induced DNA lesions and suggest that their defective repair could lead to specific deficits in neuronal functions. Notably, the genome-wide distribution of TOP2B-mediated DSBs have thus far been mapped in only a few scenarios and the prevalence of such DSBs in other cells, especially other differentiated cell types remains to be determined (Bunch et al., 2015; Madabhushi et al., 2015; Stott et al., 2021). While all cell types are likely to incur lesions resulting from accidental topoisomerase poisoning, the observations that defective repair of lasting TOP2B-mediated DSBs in genes crucial for synaptic plasticity, learning, and memory formation could explain how mutations in factors that repair topoisomerase-induced DNA lesions could result in primarily neurological phenotypes.

## Repair of Topoisomerase-Mediated DNA Damage

**FIGURE 2:**
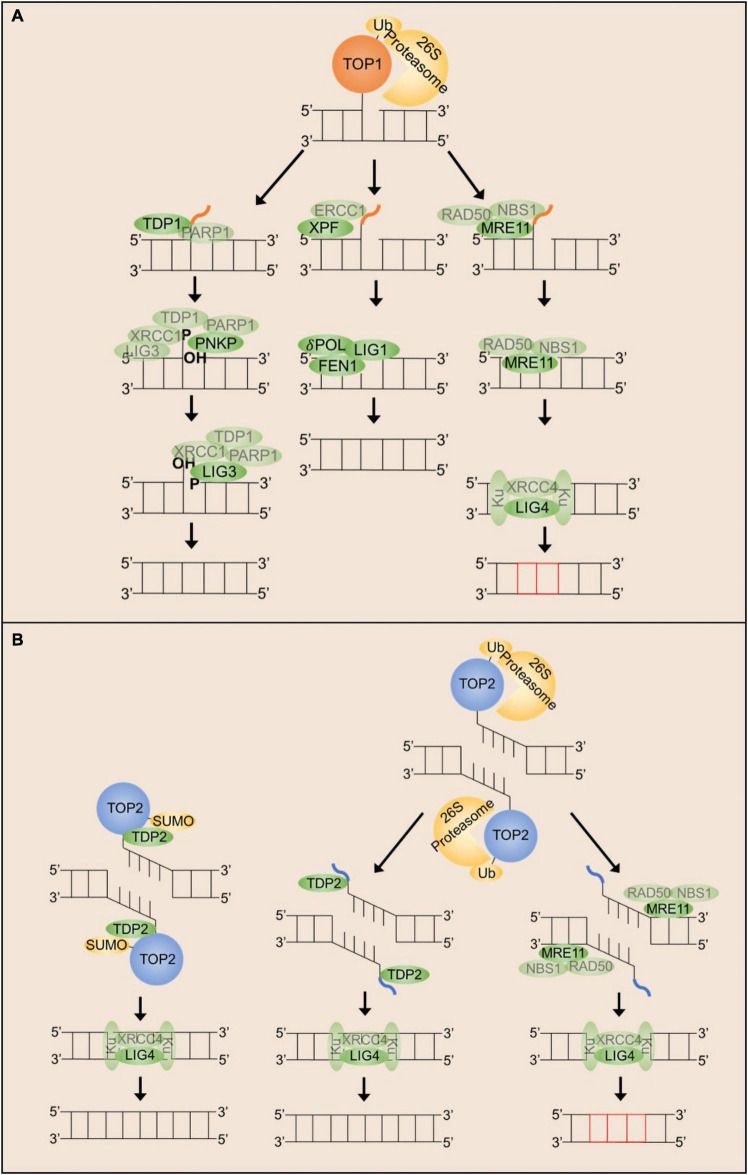
Mechanisms that can repair stalled TOP1 and TOP2 cleavage complexes in post-mitotic neurons. **(A)** TOP1ccs can be resolved by either TDP1, XPF/ERCC1 or MRN dependent mechanisms. Each of these mechanisms appear to require proteasomal degradation of the TOP1 adduct. **(B)** TOP2ccs can be resolved either by TDP2 or by MRN dependent mechanisms. Each of these mechanisms can be utilized following proteasomal degradation of the TOP2 adduct, but TDP2 can also facilitate direct hydrolysis of the phospho-tyrosyl bond.

As highlighted above, topoisomerases are likely a significant source of endogenous DNA damage. Accordingly, mammalian cells have evolved several redundant mechanisms capable of processing topoisomerase-mediated DNA lesions. In general, the resolution of TOP1 and TOP2 covalent complexes follows a similar scheme (Figure 2). The topoisomerase adduct is recognized and marked for proteasomal degradation, allowing enzymes to access and cleave the covalent phosphotyrosyl bond or the adjacent DNA, producing free DNA ends that can be repaired by canonical SSB or DSB repair machineries. Despite our knowledge of redundant pathways, the mechanisms that facilitate repair pathway choice for TOP1 and TOP2-induced DNA breaks remain elusive.

### Repair of Topoisomerase I-Mediated DNA Damage

TOP1ccs consist of a SSB with a free 5′-hydroxyl terminus and a 3′-phosphate terminus covalently attached to the TOP1 active site tyrosine (Champoux, 2003; Pommier et al., 2010). Although a full picture of how stalled TOP1ccs are distinguished from transient catalytic intermediates is incomplete, recent research suggests a prominent role for TOP1 post-translational modification and degradation. Following treatment with CPT, stalled TOP1ccs are substrates for ubiquitination, small ubiquitin-like modification (SUMOylation), and phosphorylation (Desai et al., 2003; Lin et al., 2008; Comeaux and van Waardenburg, 2014; Sun et al., 2020a). These modifications promote the recognition of TOP1ccs by the 26S-proteasome to facilitate TOP1 degradation (Desai et al., 2003; Lin et al., 2008). In neurons specifically, proteasomal degradation is shown to be dependent on ATM, although this requirement is independent of ATM kinase activity (Katyal et al., 2014). Following TOP1cc recognition and degradation, resolution of TOP1ccs proceeds via one of two mechanisms in mammalian cells: TOP1 excision by TDP1 or TOP1 excision by endonucleases.

#### TOP1 Excision by TDP1

TDP1 (tyrosyl-DNA phosphodiesterase 1) is a phospholipid hydrolyzing enzyme first identified in 1996 as the enzyme that hydrolyzes the covalent bond between the TOP1 active site tyrosine and the 3′ end of the DNA backbone (Yang et al., 1996; Pouliot et al., 1999). Sequence alignments and crystal structures indicate TDP1 is a member of the phospholipase D family of enzymes (Stuckey and Dixon, 1999; Interthal et al., 2001; Davies et al., 2002; Flett et al., 2018). In addition to phosphotyrosyl bonds, TDP1 is able to hydrolyze a variety of other substrates including 3′-phosphoglycolate and 3′-deoxyribose ends, common products of oxidative or radiation DNA damage (Inamdar et al., 2002; Interthal et al., 2005a; Zhou et al., 2009). TDP1-deficient mammalian cells are hypersensitive to CPT as well as oxidative damage, ionizing radiation, and alkylation DNA damage (Murai et al., 2012). The sensitivity of TDP1-deficient cells to other damaging agents is likely due to the propensity of these lesions to trap TOP1ccs (Daroui et al., 2004; Sordet et al., 2004) rather than the requirement of TDP1 for the resolution of these lesions since APE1 appears to be the preferred enzyme for cleavage of 3′-phosphoglycolate and 3′-deoxyibose ends (Parsons et al., 2004, 2005; Harris et al., 2009). TDP1 also possesses weak 5′-phosphodiesterase activity *in vitro* but TDP1 deficient cells are only sensitive to etoposide at extremely high doses suggesting it is not a strongly preferred mechanism for the resolution of TOP2ccs *in vivo* (Nitiss et al., 2006; Murai et al., 2012). Early studies showed TDP1 catalytic efficiency in processing TOP1ccs decreases with the size of the TOP1 adduct, suggesting TOP1 degradation is required for efficient TDP1 activity (Debéthune et al., 2002).

TDP1 hydrolysis proceeds via a two-step reaction. In the first step, TDP1 catalyzes the metal-independent hydrolysis of the 3′-phosphotyrosyl bond forming a TDP1 covalent complex intermediate via covalent linkage between the active site H263 and the 3′-phosphate DNA end. The second step occurs when the H493 residue of TDP1 activates a water molecule to hydrolyze the phosphohistidine bond and release TDP1 from the DNA end (Interthal et al., 2001; Sun et al., 2020b). Upon completion of the catalytic cycle, TDP1 generates a 3′-phosphate end that must be hydrolyzed to a 3′-hydroxyl prior to re-ligation (Interthal et al., 2001; Plo et al., 2003). In mammalian cells, PNKP functions as a phosphatase and a kinase to process both the 3′-phosphate left by TDP1 and the 5′-hydroxyl left by the TOP1cc to generate the 3′-hydroxyl and 5′-phosphate ends required for DNA ligase activity (Kalasova et al., 2020). TDP1-mediated excision of TOP1 is facilitated by a basal interaction between TDP1 and PARP1 (Zhang et al., 2011; Das et al., 2014). Upon recognition of a stalled TOP1cc, PARP1 binds to the free 5′-hydroxyl end where it catalyzes the PARylation of TDP1 and various other proteins (Das et al., 2014). TDP1 PARylation stabilizes TDP1 binding to the DNA and facilitates the recruitment of the scaffold protein XRCC1, which in turn recruits PNKP, β-polymerase, and DNA ligase III (Caldecott et al., 1994; Vidal et al., 2001; Whitehouse et al., 2001; Plo et al., 2003; Das et al., 2014). This multimeric protein complex constitutes the basic machinery necessary for short-patch base excision repair.

Multiple studies suggest TDP1 post-translational modification is an important determinant of repair pathway choice. In addition to PARylation, TDP1 is phosphorylated by the DDR kinases DNA-PK and ATM and is also shown to be SUMOylated in response to transcription-associated TOP1ccs in neurons (Das et al., 2009; Chiang et al., 2010; Hudson et al., 2012). As with PARylation, SUMOylation and phosphorylation of TDP1 also facilitate the recruitment and retention of TDP1 at sites of DNA damage, supporting a model in which N-terminal modification of TDP1 determines whether TOP1ccs are repaired by either TDP1 or an alternative mechanism (Das et al., 2014; Brettrager et al., 2019).

#### TOP1 Excision by Endonucleases

Existing data supports a model in which TDP1 modification serves as a molecular determinant of repair pathway choice. Yet the precise conditions that favor specific posttranslational modification of TDP1 are less clearly understood. For instance, it is unknown whether all sites of TOP1ccs are equally accessible to TDP1, PARP1, and DNA damage response (DDR) kinases. Differences in these contexts could favor the resolution of TOP1ccs using alternative mechanisms. Several lines of evidence support the existence of alternative pathways for the resolution of TOP1ccs. Endonucleases implicated in the repair of TOP1ccs formed at stalled replication forks have been reviewed previously (Pommier et al., 2003; Mei et al., 2020) and will not be discussed here given the post-mitotic nature of neurons, although such repair mechanisms could be important during neural development. The two main endonuclease complexes shown to facilitate the replication-independent resolution of stalled TOP1ccs are the XPF/ERCC1 endonuclease complex and the MRN endonuclease/exonuclease complex.

XPF/ERCC1 is thought to serve as an alternative to TDP1 for the resolution of TOP1-mediated SSBs (Liu et al., 2002; Zhang et al., 2011; Takahata et al., 2015). XPF/ERCC1 can resolve mammalian TOP1ccs *in vitro*, and XPF/ERCC1 along with other components of the nucleotide excision repair (NER) machinery co-localize with some CPT-induced TOP1ccs *in vivo* (Takahata et al., 2015). Further *in vitro* characterization supports a mechanism by which XPF, stabilized by ERCC1, excises the TOP1 adduct and several adjacent nucleotides, and the resulting gap is filled by NER/long-patch SSBR machinery including FEN1, δ-polymerase, and ligase I (Takahata et al., 2015). Cells deficient for XPF, ERCC1, or FEN1 are mildly sensitive to CPT compared to WT cells and are hypersensitive to CPT when combined with the loss of TDP1, suggesting that XPF/ERCC1 could be an alternative repair mechanism for TOP1ccs (Zhang et al., 2011). Given the role of these proteins in transcription-coupled NER (TC-NER), it is hypothesized that this pathway may serve as a primary or backup mechanism to ensure proper resolution of transcription-associated TOP1ccs (Zhang et al., 2011). However, this hypothesis has not been fully elucidated.

As discussed above, TOP1-mediated SSBs can be converted into DSBs if they occur in the vicinity of other DNA strand breaks or are associated with transcription (Sordet et al., 2009). The MRN complex (MRE11/RAD50/NBS1) is hypothesized to serve as an alternative pathway for the resolution of TOP1-mediated DSBs. MRE11 endonuclease/exonuclease activity was previously believed to commit cells to DSB repair by homologous recombination (HR), but it has since been shown that MRE11 nuclease activity can also facilitate repair by non-homologous end joining (NHEJ) (Shibata et al., 2014), and such a mechanism could be utilized in post-mitotic neurons. The nuclease activity of MRE11 alone is sufficient to process 3′-phosphotyrosyl bonds *in vitro* (Sacho and Maizels, 2011) and all three components of the MRN complex co-localize with TOP1ccs *in vivo* (Robison et al., 2005; Gorodetsky et al., 2007). Localization of this complex at TOP1ccs and activation of MRE11 endonuclease activity could facilitate the excision of the TOP1 adduct (Deshpande et al., 2016). Removal of the TOP1 adduct could then allow end-recognition by the Ku 70/80 heterodimer, and Ku end binding in turn recruits the XRCC4-LIG4 complex to facilitate repair via NHEJ (Davis and Chen, 2013). However, the prevalence and significance of TOP1cc processing for mammalian cells is unclear. Synthetic lethality has been reported between *tdp1* and *mre11* in yeast, however, mice lacking either both *Tdp1* and *Nbs1* or *Tdp1* and *Mre11* in the brain showed no obvious phenotypes (Katyal et al., 2014). A direct examination of TOP1ccs in these mice could clarify the importance of MRN complex in this process.

### Repair of Topoisomerase II-Mediated DNA Damage

TOP2ccs consist of a double strand break (DSB) with a free 3′-hydroxyl terminus and a 5′-phosphate terminus covalently attached to the TOP2 active site tyrosine (Champoux, 2003). Mechanisms responsible for the detection and resolution of TOP2ccs are even more ill-defined than for TOP1ccs. TOP2ccs are similarly substrates for ubiquitination, SUMOylation, and phosphorylation, but the physiological role for these post-translational modifications remains unclear. It has been hypothesized that the first step for recognition and resolution of TOP2ccs involves ubiquitination and subsequent degradation of the TOP2 adduct (Mao et al., 2000, 2001; Zhang et al., 2006; Gao et al., 2014), but more recent studies have suggested that degradation is not always required for TOP2 excision (Schellenberg et al., 2017). TOP2 SUMOylation is also thought to be an important modification to distinguish stalled TOP2ccs from normal catalytic intermediates and recruit necessary repair factors (Ryu et al., 2010; Schellenberg et al., 2017; Lee et al., 2018; Sun et al., 2020a). As with TOP1ccs, TOP2ccs are resolved via two main mechanisms in mammalian cells: TOP2 excision by either TDP2 or TOP2 excision by endonucleases.

#### TOP2 Excision by TDP2

TDP2 (tyrosyl-DNA phosphodiesterase 2) is a highly conserved member of the endonuclease/exonuclease/phosphatase (EEP) family of enzymes. Unlike TDP1, early characterization of TDP2 was not focused on its role in the resolution of TOP2ccs. TDP2 (also known as EAPII/TTRAP) was previously described as a promiscuous binding protein involved in several signaling transduction cascades including TNF-TNFR, TGFβ, and MAPK (Li et al., 2011). The phosphodiesterase activity of TDP2 was initially discovered through genetic screens designed to identify proteins that can resolve TOP1ccs in the absence of TDP1 (Ledesma et al., 2009). Although TDP2 is capable of processing 3′-phosphotyrosyl linkages with low efficiency, TDP2 deficient cells are not sensitive to CPT treatment suggesting TDP2 is not readily used for the repair of TOP1ccs (Zeng et al., 2012). Further characterization uncovered high specificity and efficiency for the hydrolysis of 5′-phosphotyrosyl bonds (Gao et al., 2012; Schellenberg et al., 2012, 2016; Shi et al., 2012), and TDP2 was shown to be the main 5′-phosphodiesterase in mammalian cells with a prominent role in the resolution of TOP2ccs (Ledesma et al., 2009; Zeng et al., 2011). TDP2-deficient cells are hypersensitive to ETP treatment and show elevated levels of γH2AX and higher levels of chromosome translocations, but they do not accumulate endogenous TOP2ccs, indicating the existence of other redundant mechanisms to process TOP2ccs (Gómez-Herreros et al., 2014; Schellenberg et al., 2017; Lee et al., 2018). However, these results have not been verified in post-mitotic neurons.

TDP2 hydrolysis proceeds via a metal dependent S2N displacement reaction (Adhikari et al., 2012; Gao et al., 2012; Schellenberg et al., 2012). TDP2 recognizes and binds both the protein and DNA component of the TOP2cc via an induced-fit binding mechanism that allows for proper positioning of a water molecule and magnesium ion for activation of the catalytic site and hydrolysis, respectively (Schellenberg et al., 2012). Unlike TDP1, TDP2 catalysis does not involve the generation of a covalent intermediate and produces 5′-phosphate ends that are compatible for direct ligation by the NHEJ proteins Ku and ligase IV without the need for further end processing (Gó Mez-Herreros et al., 2013; Schellenberg et al., 2016). TDP2 is initially unable to access the phospho-tyrosyl bond that is buried in the proteinaceous shell of the TOP2-DNA adduct. TOP2 is shown to be degraded by the proteasome following treatment with etoposide (Mao et al., 2001) and TDP2 activity is accelerated on free phospho-tyrosyl DNA ends compared to intact TOP2ccs (Gao et al., 2012; Schellenberg et al., 2016), suggesting proteasomal degradation of the TOP2 adduct is one strategy for facilitating TDP2 hydrolysis. More recently, it was shown that the TOP2 adduct can undergo conformational change to allow TDP2 access to the phospho-tyrosyl bond without the need for proteolytic degradation through interaction with the ZATT SUMO ligase (Schellenberg et al., 2017).

Recruitment of TDP2 potentially occurs via two different mechanisms depending on the mechanism used to process or remodel the TOP2 adduct. If TOP2 is marked with ubiquitination for subsequent proteasomal degradation, this could recruit TDP2 via it’s N-terminal ubiquitin binding (UBA) domain (Rao et al., 2016; Schellenberg et al., 2020). Binding of ubiquitin to the UBA domain of TDP2 does stimulate its phosphodiesterase activity *in vitro* and TDP2 does bind to a pool of ubiquitinated proteins *in vivo*, but this pool of ubiquitinated proteins does not appear to include TOP2 (Rao et al., 2016; Schellenberg et al., 2020). Still, there is conflicting evidence for whether deletion of the UBA domain confers hypersensitivity to etoposide depending on the cell-type used, suggesting potential cell-type specific roles for the UBA domain of TDP2 that may or may not be independent of its role in the recruitment of TDP2 to stalled TOP2ccs (Rao et al., 2016; Schellenberg et al., 2020). Alternatively, SUMOylation of the TOP2 adduct by ZATT to facilitate TOP2 remodeling could recruit TDP2 via its non-canonical SUMO binding (split-SIM) domain (Schellenberg et al., 2017). TDP2 forms a stable complex with TOP2 and ZATT *in vivo*, and TOP2 SUMOylation and TDP2 recruitment is markedly diminished in ZATT-deficient cells (Schellenberg et al., 2017). TDP2-deficient cells fail to process a specific pool of SUMOylated TOP2ccs (Schellenberg et al., 2017; Lee et al., 2018), further supporting a model by which TOP2 SUMOylation marks stalled TOP2ccs for processing by TDP2.

#### TOP2 Excision by Endonucleases

Similar to its role in the resolution of TOP1ccs, the MRN complex functions in parallel to TDP2 for the excision of stalled TOP2ccs. The nuclease activity of MRE11 excises the TOP2 adduct and several adjacent nucleotides to produce free DNA ends that can be re-ligated via NHEJ machinery. The MRN complex is likely more important for the resolution of TOP2ccs than TOP1ccs, as MRE11-deficient cells accumulate endogenous TOP2ccs and are hypersensitive to ETP, but do not accumulate TOP1ccs or display CPT sensitivity (Lee et al., 2012; Hoa et al., 2016). MRE11-deficient lymphoblastoid cells display similar delays in DSB repair following treatment with ETP as TDP2-deficient cells, and loss of both enzymes has an additive effect that is similar to loss of LIG4 (Hoa et al., 2016). Additionally, deficits in TOP2cc resolution and repair in MRE11-deficient cells can be rescued by overexpression of TDP2 (Hoa et al., 2016). These data suggest both pathways contribute to the resolution of TOP2ccs by NHEJ. Interestingly, MRE11 was shown to selectively facilitate the resolution of TOP2Accs, but not TOP2Bccs *in vitro* (Lee et al., 2012). Whereas proliferating vertebrate cells express two TOP2 isoforms, TOP2A and TOP2B, post-mitotic cells, such as neurons, only express Top2B (Madabhushi, 2018). These results therefore raise the possibility that MRE11 might not be effective at resolving TOP2ccs in neurons. However, endogenous TOP2ccs were shown to accumulate in the mouse brain in *Nbs1*^–/–^ mutants, suggesting that the MRN complex could in fact be important for their resolution *in vivo* (Hoa et al., 2016). Future studies that directly assess the effects of MRN activity on TOP2ccs in neurons should clarify this issue.

## Topoisomerase-Mediated DNA Damage and Neurological Disease

The consequences of congenital deficits in DNA repair processes vary depending on the nature of the signaling pathways that are perturbed, ranging from immune deficiency, photosensitivity, and cancer (Tiwari and Wilson, 2019). However, one thing that nearly all DNA repair deficiency syndromes have in common is profound effects on the central nervous system, often manifesting in neurodegeneration (Mckinnon, 2013; Madabhushi et al., 2014; Alt and Schwer, 2018; Abugable et al., 2019). In fact, defective DNA repair has recently been linked to the progression of common neurodegenerative diseases such as Alzheimer’s disease and Parkinson’s disease (Thadathil et al., 2019). Despite an abundance of evidence supporting this relationship, it is still unclear how disruption of a process that is crucial for the survival of all cells can be selectively toxic to post-mitotic neurons.

In general, neurological symptoms that result from mutation to DNA repair factors can provide clues about which mechanisms are preferred in the brain compared to other tissues. Many inherited disorders of DNA repair have been identified with one shared presentation—cerebellar degeneration leading to progressive ataxia (Taroni and DiDonato, 2004; Paulson and Miller, 2005; Gilmore, 2014; McKinnon, 2014). Attempts to understand the relationship between DNA repair deficits and ataxic symptoms have largely focused on biochemical characterization of repair pathways without a heavy focus on understanding the source of the lesions and why these sources of damage are uniquely problematic in the nervous system. The identification of two inherited ataxias exhibiting exclusively neurological symptoms that are caused by mutations in TDP1 (SCAN1) and TDP2 (SCAR23) point to the potential importance of topoisomerase-mediated DNA damage in the brain (Zagnoli-Vieira and Caldecott, 2020).

### Disorders of TOP1cc Resolution: Spinocerebellar Ataxia With Axonal Neuropathy

Initial evidence that deficits in TOP1cc repair can contribute to the development of neurological phenotypes was observed for SCAN1, an autosomal recessive syndrome characterized almost exclusively by neurological deficits (Takashima et al., 2002; El-Khamisy et al., 2005; El-Khamisy and Caldecott, 2007; Walton et al., 2010). SCAN1 patients display progressive ataxia, cerebellar degeneration, and peripheral neuropathy with average onset occurring in the second decade of life, suggesting SCAN1 is a neurodegenerative rather than neurodevelopmental syndrome (Takashima et al., 2002). The cause of SCAN1 was identified as a mutation in TDP1, and SCAN1 cells accumulate endogenous TOP1ccs, are hypersensitive to CPT, and mildly sensitive to oxidative damage (Takashima et al., 2002; Interthal et al., 2005b; Miao et al., 2006; Katyal et al., 2007). TDP1-deficient cells are expectedly deficient for SSB repair but not DSB repair, suggesting TOP1-mediated DSBs are not a major source of pathology in SCAN1 (Katyal et al., 2007). Endogenous accumulation of TOP1ccs in TDP1-deficient cells is prevented by treatment with transcription inhibitors or antioxidant agents (Katyal et al., 2014), suggesting transcription and oxidative stress are major contributors to steady state levels of TOP1ccs that become pathological in SCAN1.

Mouse models of SCAN1 exhibit similar molecular phenotypes including accumulation of TOP1ccs and reduced SSB repair following treatment with CPT or oxidative damage, and also exhibit age-related cerebellar atrophy (Hirano et al., 2007; Katyal et al., 2007; Hawkins et al., 2009). However, SCAN1 mouse models do not recapitulate the behavioral phenotypes observed in human patients, and no age-related increase in ataxia symptoms has been observed (Hirano et al., 2007; Katyal et al., 2007; Hawkins et al., 2009). Given that the mouse models employed are TDP1-null mice rather than TDP1-mutant mice, it may be important to understand the pathology of the specific mutation underlying SCAN1. The causal TDP1 mutation underlying SCAN1 (H493R) inhibits the second step of the TDP1 reaction mechanism that resolves the TDP1-DNA catalytic intermediate (Interthal et al., 2005b; He et al., 2007; Cuya et al., 2016). Therefore, SCAN1 cells are thought to accumulate TDP1ccs in addition to TOP1ccs. The accumulation of TDP1ccs further impedes the recognition and resolution of these lesions by alternative mechanisms that typically respond to TOP1cc formation, and thus TDP1 mutation could have more severe consequences than total loss of TDP1 expression. Preliminary evidence supporting this idea is observed in yeast cells. TDP1-null yeast cells are not sensitive to CPT alone and only mildly sensitive to increased levels of TOP1ccs, but yeast expressing TDP1^H493R^ are hypersensitive to CPT treatment (He et al., 2007).

### TOP1cc Resolution in Other DNA Repair Disorders

The first syndrome that provided a direct link between DNA damage and neurological abnormalities is ataxia telangiectasia (AT), an inherited syndrome caused by mutation to ATM (Mckinnon, 2012; Amirifar et al., 2019). The most common features of AT are cerebellar degeneration and progressive ataxia, but AT patients also exhibit some extra-neurological symptoms, including sensitivity to ionizing radiation, genome instability, and increased cancer incidence. The ubiquitous nature of ATM in modulating responses to DNA damage makes it difficult to determine the molecular causes of the neurological phenotypes observed in AT. Several lines of evidence suggest dysregulated TOP1cc resolution is a contributing factor. ATM-deficient mouse neurons accumulate high levels of endogenous TOP1ccs, although slightly lower than what is observed for TDP1-deficient cells, with the highest accumulation observed in the cerebellum at 1 year of age (Katyal et al., 2014). ATM-deficient embryonic mice systemically treated with CPT exhibit apoptosis that is nearly exclusively confined to the central nervous system, highlighting the importance of ATM in the response to TOP1-mediated lesions (Katyal et al., 2014). Intriguingly, accumulation of ATM, the MRN complex, and TOP1 in the nucleus of mature Purkinje neurons has been observed (Gorodetsky et al., 2007). Endogenous accumulation of TOP1ccs in ATM-deficient cells can be abrogated by pre-treatment with transcription inhibitors or antioxidant agents (Alagoz et al., 2013; Katyal et al., 2014), further supporting the assertion that transcription and oxidative stress are the major contributors to endogenous TOP1cc formation.

The relationship between ATM deficiency and TOP1cc accumulation is likely due to the role of ATM in facilitating proteasomal degradation of stalled TOP1ccs. ATM-deficient neurons do not exhibit CPT-induced proteasomal degradation of TOP1 and therefore accumulate endogenous TOP1ccs that include full-length TOP1 that is not degraded by the proteasome (Alagoz et al., 2013; Katyal et al., 2014). This deficit in proteasomal degradation of TOP1 in ATM-deficient cells is likely due to an observed decrease in SUMOylation and ubiquitination of TOP1 following CPT treatment which would typically signal for proteasomal degradation (Katyal et al., 2014). Interestingly, the role of ATM in facilitating proteasomal degradation appears to be independent of ATM kinase activity as inhibiting ATM kinase activity does not result in decreased proteasomal degradation of TOP1 or accumulation of TOP1ccs (Katyal et al., 2014). More work must be done to fully elucidate the biochemical mechanism by which ATM promotes the SUMO/ubiquitin-mediated degradation of stalled TOP1ccs to facilitate SSB repair, but current evidence suggests pathological TOP1cc accumulation contributes to the neurological abnormalities observed in AT.

Mutation to XRCC1 similarly leads to the accumulation of endogenous TOP1ccs and cerebellar degeneration, though human patients harboring these mutations are not well characterized (Lee et al., 2009; Katyal et al., 2014; Hoch et al., 2016). Mutation to PNKP is the underlying cause of microcephaly with early onset seizures (MCSZ) and cerebellar degeneration in ataxia oculomotor apraxia-4 (AOA4) (Kalasova et al., 2019). PNKP-deficient cells do show reduced SSB repair capacity following treatment with CPT, but the role of aberrant TOP1cc formation in PNKP-induced pathology has not been elucidated (Kalasova et al., 2020). In contrast, preliminary characterization of yet another disorder with ataxia and cerebellar degeneration known as ataxia oculomotor apraxia-1 (AOA1) suggests aberrant TOP1cc formation is not a primary driver of AOA1 pathology. AOA1 is caused by mutation to aprataxin (APTX), a 5′-AMP processing enzyme that resolves failed ligation intermediates (Seidle et al., 2005; Ahel et al., 2006). Whereas APTX-deficient cells exhibit deficits in SSB repair following oxidative damage they are not sensitive to CPT treatment and do not accumulate endogenous TOP1ccs (El-Khamisy et al., 2009; Reynolds et al., 2009; Katyal et al., 2014). Clearly there is some heterogeneity in the causes of cerebellar degeneration and ataxia. However, disorders that involve mutations to core SSB repair machinery do seem to involve aberrant TOP1cc formation, highlighting the relevance of TOP1-mediated lesions in the brain.

### Disorders of TOP2cc Resolution: Spinocerebellar Ataxia Autosomal Recessive 23

While it has long been established that deficits in DSB repair lead to the development of neurological abnormalities (Alt and Schwer, 2018), the identification of patients with SCAR23 indicated that topoisomerase-induced DNA damage could be an important relevant lesion for neurological abnormalities. SCAR23 is an autosomal recessive syndrome that is characterized by treatment-resistant epilepsy, progressive ataxia, and cerebellar degeneration (Gó Mez-Herreros et al., 2013; Gómez-Herreros et al., 2014). SCAR23 patients also display a later age of onset than other inherited ataxias, with symptom severity increasing during the second decade of life, suggesting SCAR23 is also a degenerative rather than developmental disorder (Gómez-Herreros et al., 2014; Zagnoli-Vieira et al., 2018; Ciaccio et al., 2019; Errichiello et al., 2020). The underlying cause of SCAR23 are mutations within TDP2, and SCAR23 cells are deficient for the resolution of stalled TOP2ccs and are hypersensitive to ETP (Gómez-Herreros et al., 2014). Several disease-causing mutations in TDP2 have been identified, each resulting in truncated mRNA expression and nonsense-mediated decay (Gómez-Herreros et al., 2014; Zagnoli-Vieira et al., 2018; Ciaccio et al., 2019; Errichiello et al., 2020). Like SCAN1, mouse models of SCAR23 recapitulate the molecular and cellular phenotypes of human patients but do not display similar behavioral abnormalities such as ataxia or increased seizure propensity (Gómez-Herreros et al., 2014).

Recent research suggests an important role for TOP2cc resolution by TDP2 during transcription. TDP2-deficient neurons show significant delays in recovery of transcription following treatment with ETP (Gómez-Herreros et al., 2014). Genome-wide expression profiling show over 100 genes are downregulated in TDP2-deficient neurons compared to WT neurons, and ∼half of these genes are known to be associated with the etiology of seizures/epilepsy, ataxia, and cognitive development (Gómez-Herreros et al., 2014). Intriguingly, genes that are differentially expressed in TDP2-defficient cells are significantly longer than the average length of all transcripts analyzed, suggesting long genes are most heavily affected (Gómez-Herreros et al., 2014). The observed changes in the expression of long genes are in line with other recent studies showing topoisomerase inhibition results in a length-dependent impairment in gene expression in post-mitotic neurons (Zylka et al., 2015), which will be discussed in more detail below.

### TOP2cc Resolution in Other DNA Repair Disorders

Existing molecular and behavioral characterization for the relative importance of TOP2cc resolution mechanisms in the brain is far less clear than what has been described for TOP1cc resolution mechanisms. In addition to TDP2 deficiency associated with SCAR23, MRE11 deficiency also results in exclusively neurological phenotypes, including cerebellar degeneration and progressive ataxia, and described as ataxia telangiectasia-like disorder (ATLD) (Taylor et al., 2004). Hypomorphic mutations in MRE11 cause ATLD and these mutations do not disrupt nuclease activity but impede proper formation of the MRN complex and MRE11 subcellular localization (Stewart et al., 1999; Delia et al., 2004). As mentioned above, the available data paints a complicated and incomplete picture of the role of MRE11 for the resolution of TOP2ccs in proliferating cells vs. post-mitotic neurons. Hypomorphic MRE11 mutations result in exclusively neurological symptoms, suggesting MRE11 does in fact play an important role in the brain (Taylor et al., 2004). However, an important unanswered question in this regard is whether failure to resolve TOP2ccs could contribute to the pathology of ATLD or whether disease phenotypes arise from a more general requirement of MRE11 in DNA repair.

In addition to a potential role for aberrant TOP1cc formation in the pathology of AT, there is a likely contribution of TOP2cc formation as well. ATM is generally thought to be important for general DSB recognition and signaling, but it has been shown to be dispensable for the repair of most DSBs in proliferating cells (Caron et al., 2015). Instead, ATM is thought to be required for the resolution of “complex” DSBs that contain blocked DNA ends such as those generated by TOP2ccs (Alvarez-Quilon et al., 2014; Clouaire et al., 2017). The inability of ATM-deficient cells to resolve TOP2ccs could be particularly important for neuronal pathology of AT since neuronal activity is known to result in the formation of stalled TOP2ccs (Madabhushi et al., 2015).

### Toward an Understanding of Topoisomerase-Mediated DNA Damage in the Maintenance of Neuronal Health and the Development of Neurological Disease

As mentioned above, the precise underlying mechanisms by which defective repair of topoisomerase-mediated lesions lead to neurological disorders are still unclear. However, recent studies provide several insights into this issue. Many of the cytotoxic effects of topoisomerase-induced lesions have been linked to their interactions with DNA replication and transcription. Because neurons are post-mitotic, it is thought that interference with gene transcription and gene activity patterns could explain disease-related neuropathologies. In this regard, an examination of gene transcription in neurons revealed that the loss of both TOP1 and TOP2B primarily affect the transcription of long genes (>100 kb) in postmitotic neurons (King et al., 2013). Specifically, treatment with either TOP1 and TOP2 inhibitors or silencing their expression in primary cortical neurons causes the downregulation of extremely long genes, with a strong negative correlation between gene length and change in gene expression (King et al., 2013; Gokoolparsadh et al., 2017; Fragola et al., 2020). These findings provide us with one potential explanation for why neurons are particularly vulnerable to perturbations in topoisomerase activity. When examining genome-wide expression across all tissues, the longest transcripts are strongly enriched for neuronal genes (Gabel et al., 2015; Lopes et al., 2021). Gene ontology analysis of the longest genes in the mammalian genome is highly enriched for neuronal terms related to signaling molecules, ion channels, receptors, and synaptic transmission molecules that govern neuronal excitability and connectivity (Gabel et al., 2015; Mabb et al., 2016; Sugino et al., 2019; Lopes et al., 2021).

**FIGURE 3:**
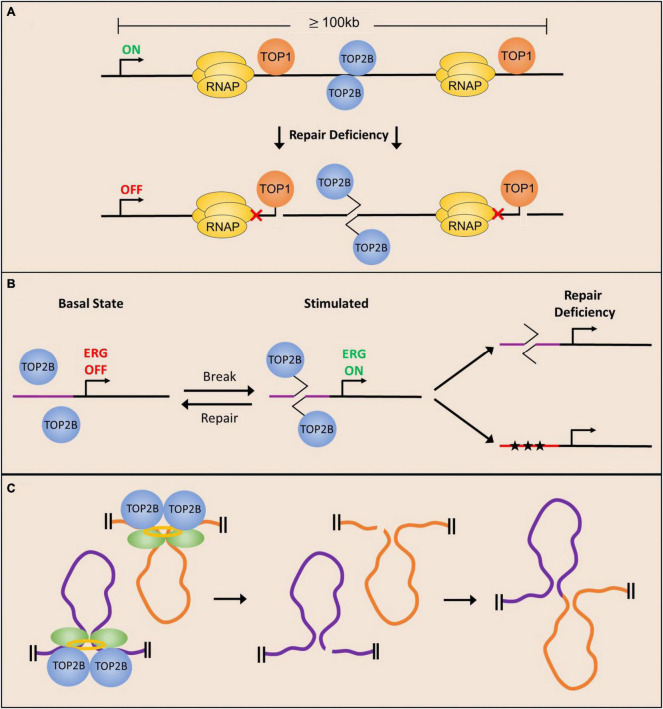
Potential mechanisms underlying the development of neurological phenotypes due to deficient repair of topoisomerase-mediated lesions. **(A)** Long neuronal genes require topoisomerase activity to facilitate their expression and are therefore more likely to incur stochastic cleavage complex formation. Deficient repair of stalled cleavage complexes results in polymerase pausing and downregulation of long gene expression. **(B)** Neuronal stimulation facilitates the formation of TOP2ccs in the promoters of ERGs to facilitate their expression, and repair of the TOP2-mediated break restores basal conditions. Deficient repair can either cause delayed break repair or mutagenesis (stars) resulting in dysregulation of ERG expression. **(C)** TOP2-mediated DNA DSBs at chromatin loop anchors could lead to altered gene activity patterns in neurons and compromise neuronal functions.

Although genome-wide expression data for DNA repair deficient neurons is sparse, loss of TDP2 or ERCC1 expression is shown to result in a length-dependent downregulation of gene expression (Gómez-Herreros et al., 2014; Vermeij et al., 2016), alluding to a role for the expression of long genes in disease pathology. Genes that are downregulated are shown to be preferentially bound by TOP2B in WT neurons and are more likely to incur DNA breaks, which likely explains the downregulation of these genes in a repair-deficient cell (Tiwari et al., 2012; Gómez-Herreros et al., 2014; Vermeij et al., 2016; Wei et al., 2016). These long neuronal genes are generally more highly expressed at later points in neuronal development (Okaty et al., 2009), potentially contributing to the neurodegenerative aspects of many DNA repair deficient syndromes. Additionally, the magnitude of expression of long genes is up to 10-fold higher in the cerebellum and cerebral cortex compared to other tissues (Gabel et al., 2015), and these same tissues are most highly impacted by loss of TDP1 or TDP2. Taken together, these data suggest that topoisomerase activity is especially important for the expression of long genes and that the accumulation of TOP1ccs and TOP2ccs could preclude the expression of genes important for synaptic transmission and contribute to disease pathology (Figure 3A). While TOP1 and TOP2B activity is required for the expression of long genes, it should be noted that the precise roles of TOP1 and TOP2ccs in this regard are still ill-defined. On the one hand, the observation that downregulation of either TOP1 or TOP2B cause also cause a reduction in the expression of long genes suggests that accumulation of torsional stress in the absence of topoisomerases could stall elongating RNA polymerases and prevent gene transcription independent of lesion formation. On the other hand, the observation that long genes are also downregulated in mutants that show defects in the repair of topoisomerase-DNA cleavage complexes suggests that the accumulation of TOP1 and TOP2ccs could be an important mechanism of transcription inhibition. A more direct examination of whether TOP1 and TOP2ccs accumulate within the gene bodies of long genes in neurons could help clarify this issue.

In addition to being important for the transcription of long genes, recent observations that describe the enrichment of TOP2B-mediated DSBs within the promoters of ERGs and other stimulus-responsive genes following neuronal activity suggest that changes in the repair of activity-induced DSBs could have an impact on neuronal activity-dependent transcription patters and lead to the development of neurological disorders (Madabhushi and Kim, 2018). In fact, disruptions in neuronal activity-dependent gene transcription programs have been linked to numerous disorders, including intellectual disability and autism spectrum disorders (Ebert and Greenberg, 2013) and it is tempting to speculate that many of the phenotypes in SCAR23 patients could arise from the defective repair of activity-induced DSBs generated by TOP2B (Figure 3B).

Animal behavior is shaped by a lifetime of experiences that involve repeated stimulation of neurons in relevant brain areas and neurons specialize in integrating information from environmental cues to develop adaptive behaviors. While exposure to new sensory stimuli affects neurons on many levels, experience driven changes to genome organization establish gene activity patterns that enable animals to form long-term memories and other lasting adaptations (Su et al., 2017; Madabhushi and Kim, 2018; Yap and Greenberg, 2018; Beagan et al., 2020). While the application of high-resolution imaging and next-generation sequencing technologies has elaborated the state of epigenetic landscapes, chromatin accessibility, and chromatin looping interactions in stimulated neurons, recent studies suggest that dynamic supercoiling and topoisomerases could have a pivotal role in shaping chromatin architecture (Kim et al., 2010; Malik et al., 2014; Corless and Gilbert, 2016; Su et al., 2017; Bjorkegren and Baranello, 2018; Beagan et al., 2020). The role of topoisomerases, particularly TOP2B, could have important implications for the formation of topoisomerase-mediated DNA damage and its effects on neuronal function. Examination of TOP2B occupancy patterns in a variety of cell types and tissues, including in post-mitotic neurons has revealed that the binding of the architectural protein, CTCF, is highly enriched at genome-wide sites bound by TOP2B (Madabhushi et al., 2015; Uusküla-Reimand et al., 2016; Canela et al., 2017). Interestingly, analysis of supercoiling changes suggests that TOP2B activity could modulate DNA supercoiling at CTCF binding sites and that Top2B-mediated DSBs are concentrated at loop anchors bound by CTCF (Uusküla-Reimand et al., 2016; Canela et al., 2017). In fact, the probability of DSBs at loop anchors positively correlate with TOP2B binding and are associated with translocation breakpoint clusters that are dysregulated in various cancers (Canela et al., 2017, 2019). Together these results suggest that aberrant repair of TOP2B-mediated DNA DSBs could perturb chromatin organization and gene activity patterns, and thereby contribute to neuronal dysfunction (Figure 3C).

## Conclusion and Perspectives

Although recent studies have provided exciting new insights into the role of topoisomerases in the maintenance of genome integrity in the central nervous system and the etiology of neurological disease, there are still some barriers to fully understanding the role of topoisomerases in the progression of neurological symptoms in repair-deficient neurons. For instance, it is not fully understood whether the major deficit in TOPcc formation and repair occurs during developmental stages involving rapidly proliferating neurogenesis or occur as post-mitotic cells work to manage the cumulative effects of basal and stimulus-induced transcription. Indeed, many DNA repair-deficient syndromes exhibit profound neurodegeneration and age-related cognitive decline, but it is largely unclear if this could be due to cumulative deficits that initially arise during developmental phases that only present themselves at later stages. Determining a cause and effect relationship between the observed molecular, cellular, and behavioral phenotypes is made more difficult by the fact that animal models of most DNA repair deficient syndromes to not recapitulate the behavioral phenotypes across age. In addition, it is not fully understood why there cerebellum is so heavily impacted by DNA repair deficits. It has been observed that TOP1 protein levels are very high in Purkinje neurons (Gorodetsky et al., 2007), TOP1 activity is highest in inhibitory neurons of the cerebellum and striatum (Plaschkes et al., 2005) and high levels of TOP1ccs are observed in the cerebellum of TDP1-deficient mice (Katyal et al., 2014). However, the molecular logic for increased topoisomerase activity in these cells is not fully understood.

Highlighted throughout this review are several examples of gaps in knowledge that require attention to fully elucidate the mechanisms of topoisomerase cleavage complex formation and repair in post-mitotic neurons. Importantly, the recent discovery of TOP2ccs that are formed in response to physiological stimuli calls for an investigation of the mechanisms that regulate the cleavage-ligation of these enzymes following stimulation. Although it is well documented that stalled cleavage complexes are ubiquitinated to signal for proteasomal degradation prior to complex resolution and repair, it is still largely unclear how stalled TOPccs are distinguished from normal catalytic intermediates, and how this recognition facilitates repair pathway choice. The cell-type specificity of repair mechanisms is also poorly understood, and the relative importance of each of these pathways in post-mitotic neurons requires further investigation. Future efforts to address these knowledge gaps should provide much needed insights into understanding the contribution of topoisomerase-induced DNA damage to neurological disorders.

## Author Contributions

RM conceptualized the plan for the manuscript. MC and RM wrote the manuscript together. Both authors contributed to the article and approved the submitted version.

## Conflict of Interest

The authors declare that the research was conducted in the absence of any commercial or financial relationships that could be construed as a potential conflict of interest.

## Publisher’s Note

All claims expressed in this article are solely those of the authors and do not necessarily represent those of their affiliated organizations, or those of the publisher, the editors and the reviewers. Any product that may be evaluated in this article, or claim that may be made by its manufacturer, is not guaranteed or endorsed by the publisher.
